# mTOR and SGLT-2 Inhibitors: Their Synergistic Effect on Age-Related Processes

**DOI:** 10.3390/ijms25168676

**Published:** 2024-08-08

**Authors:** Dario Troise, Silvia Mercuri, Barbara Infante, Vincenzo Losappio, Luciana Cirolla, Giuseppe Stefano Netti, Elena Ranieri, Giovanni Stallone

**Affiliations:** 1 Nephrology, Dialysis and Transplantation Unit, Advanced Research Center on Kidney Aging (A.R.K.A.), Department of Medical and Surgical Sciences, University of Foggia, 71122 Foggia, Italy; 2 Renal Medicine and Baxter Novum, Department of Clinical Science, Intervention and Technology, Karolinska Institutet, 14152 Stockholm, Sweden; 3 Unit of Clinical Pathology, Advanced Research Center on Kidney Aging (A.R.K.A.), Department of Medical and Surgical Sciences, University of Foggia, 71122 Foggia, Italy

**Keywords:** senescence, aging, senotherapeutic strategies, senomorphic compounds, mTOR inhibitors, SGLT-2 inhibitors, inflammaging

## Abstract

The aging process contributes significantly to the onset of chronic diseases, which are the primary causes of global mortality, morbidity, and healthcare costs. Numerous studies have shown that the removal of senescent cells from tissues extends lifespan and reduces the occurrence of age-related diseases. Consequently, there is growing momentum in the development of drugs targeting these cells. Among them, mTOR and SGLT-2 inhibitors have garnered attention due to their diverse effects: mTOR inhibitors regulate cellular growth, metabolism, and immune responses, while SGLT-2 inhibitors regulate glucose reabsorption in the kidneys, resulting in various beneficial metabolic effects. Importantly, these drugs may act synergistically by influencing senescence processes and pathways. Although direct studies on the combined effects of mTOR inhibition and SGLT-2 inhibition on age-related processes are limited, this review aims to highlight the potential synergistic benefits of these drugs in targeting senescence.

## 1. Introduction

Aging is involved in the development of chronic diseases, such as cardiovascular [1], metabolic [2], renal [3], and neurodegenerative diseases [4], which are responsible for the majority of mortality, morbidity, and healthcare costs worldwide. A combination of frailty, sarcopenia, decreased physical resilience, and mild cognitive impairment are often present in older people. The occurrence of several pathological conditions simultaneously tends to increase significantly as people age [5]. The study of aging is a rapidly expanding and evolving field of research. Current research methods on aging are multidisciplinary, including genetics, after studies on longevity genes in cellular and animal models, as well as epigenetic regulation of gene expression. Moreover, the rapid advancement in molecular and cellular techniques such as RNA sequencing (RNA-Seq) and single-cell RNA sequencing (scRNA-seq), along with omics technologies (proteomics, metabolomics, and lipidomics), is creating new opportunities for aging research and discoveries of new biomarkers of aging [6]. Furthermore, machine learning algorithms are becoming essential tools for prediction and treatment investigations, given the vast complexity of the aging process [7].

Several studies have conclusively shown that the removal of senescence cells from a tissue leads to an extension of life- and healthspan and a significant reduction in the development of age-related pathologies [8]. Therefore, the development of senotherapeutic strategies has gained substantial traction. Two categories can be distinguished: senolytics drugs, which are pharmacological agents that eliminate senescent cells, and senomorphic compounds that are not be able to directly eliminate senescent cells but can counterbalance their detrimental effects [9]. Mammalian target of rapamycin (mTOR) inhibitors, such as rapamycin (RAPA) and everolimus (RAD), are considered senomorphic drugs. The serine/threonine kinase mTOR has a pivotal role in controlling signaling pathways involved in cellular growth, proliferation, motility, protein synthesis, and survival of the cells. Interestingly, the mTOR signaling pathway is constitutively active in cellular senescence and may leads to geroconversion, which represent a progression of cellular growth even when the normal cell cycle is blocked because of the presence of growth factors which stimulates cell cycle progression [10].

Sodium–glucose co-transporter-2 (SGLT-2) inhibitors belong to a category of medications designed to lower glucose levels by facilitating glucose excretion through urine. Among these, the most extensively studied SGLT-2i belong to the “-gliflozin” class, such as empagliflozin, dapagliflozin, and canagliflozin [11]. Because of their mechanism of action, they have emerged as therapeutic agents in type 2 diabetes (T2D). Further studies are trying to identify if SGLT-2i have anti-aging roles and preliminary results have focused on cellular and molecular mechanisms intertwined with the aging process and the onset of age-related diseases, such as cellular senescence and inflammaging [12,13].

This review aims to emphasize the potential synergistic effects of combining the inhibition of SGLT-2 and mTOR on the aging process.

## 2. What Is “Senescence”

### 2.1. Definition

“Cellular senescence” (or merely “senescence”) is considered one of the key characteristics of aging. It is defined as a steady, non-proliferative state in which cells enter in response to acute or chronic cellular damage and stress.

### 2.2. Influencing Factors

In humans, senescent cells accumulate in various tissues at varying rates, and it is initiated, at least partially, by the shortening of telomeres as individuals age. A telomere is a region of repetitive nucleotide sequences associated with specialized proteins at the ends of linear chromosomes. Its role is to prevent chromosomal instability. In humans, the average telomere length declines from approximately 11 kilobase at birth to less than 4 kb in old age [14,15]. In addition to telomere attrition, mitochondrial and oxidative damage, as well as nutrient imbalance, and oncogenic signaling can trigger senescence. Moreover, extracellular mediators of inflammation and fibrosis have been found to induce senescence cell phenotype [16]. The factors influencing the development of cellular senescence include chronological age, genetic and epigenetic alterations, lifestyle habits, and lifelong psychosocial influences [17]. Moreover, biological sex could influence the development of senescence. Studies on the DNA damage response have shown that women have a lower capacity for DNA repair, even though both sexes experience a similar DNA damage burden across their lifespans [18]. Furthermore, the overexpression of p53 in Drosophila increases male lifespan but reduces that of females [19]. Sex hormones have been shown to influence cellular senescence, including estrogen, testosterone, and progesterone. Specifically, estrogen has been found to negatively influence aging by promoting telomerase activity [20], suppressing the release of reactive oxygen species (ROS), thereby preventing oxidative stress [21], and upregulating the expression of proteins that prevent cell senescence, such as the Werner syndrome ATP-dependent helicase [22]. Moreover, in animal models, testosterone seems to prevent DNA damage at normal physiological concentrations [23,24,25]. It also lowers inflammatory cytokines, such as IL-6, while increasing levels of anti-inflammatory ones [26]. On the other hand, progesterone has been implicated in cellular senescence, as demonstrated by Wang and Shi, who showed that progesterone megestrol acetate drives endometrial carcinoma cell senescence, as evidenced by elevated levels of p21 and p16 [27].

### 2.3. Physiopathological Role of Senescence in Cellular Function and Structure

Senescence has a crucial role in several physiological processes, including embryogenesis, wound healing, immunosurveillance, and tumor suppression. Nevertheless, it can contribute to the pathology of various chronic diseases, including T2D, cancer, osteoarthritis, cardiovascular, neurological, and renal disease [28].

The availability of growth factors and nutrients is crucial for the activation of pathways that promote cell growth, such as mitogen-activated protein kinase (MAPK) and mTOR, and consequent induction of cyclin D1, which initiates cell cycle progression. In the absence of growth factors, cells enter in a quiescent state because MAPK/mTOR are deactivated and cyclin D1 is not induced. Nevertheless, several stress factors can induce the direct blocking of the cell cycle by triggering the expression of cyclin-dependent kinase inhibitors, such as p16 and p21, resulting in sustained cell stimulation by mTOR and MAPK and promoting unbalanced growth which freezes the cell in a hypermitogenic state, known as senescence [29]. Functional and structural changes are a peculiar feature of senescent cells. Typically, they are enlarged, vacuolated, and sometimes multinucleated with an increased number of mitochondria, partially attributed to decreased mitophagy. Functional changes include increased protein synthesis resulting in the production of misfolded proteins, reduced removal of old lysosomes, dysfunctional oxidative phosphorylation leading to the generation of ROS, and dysregulation of the activity of different proteins, including mTOR [30]. A systematic review by Tuttle et al. recently demonstrated that an elevated expression of senescence markers is observed within disease pathologies and showed that the cell cycle regulators, particularly p16, p21, and p53, are consistently upregulated in diseased human tissue samples [31].

### 2.4. High-Glucose Environment and Cellular Senescence

Glucose, among other nutrients, serves as a primary energy source for cells. A high-glucose environment has been demonstrated to increase the expression of SGLT-2 and facilitate glucose uptake and consumption, leading to the enhanced production of ROS and subsequent activation of harmful molecular pathways, such as the polyol pathway which intensifies oxidative stress, the lipid synthesis pathway resulting in the production of proinflammatory cytokines through NF-κB, the hexosamine pathway which leads to production of TGF-β, and pathways involved in the formation of advanced glycation end products (AGEs) [32]. These pathways play pivotal roles in DNA damage and p53 phosphorylation. Thus, stabilized p53 enables the transcription of the cell cycle inhibitor p21, along with other transcriptional targets, resulting in cell cycle arrest and an increase in cellular senescence [33,34].

## 3. mTOR and SGLT-2 Inhibition on Senescence Processes

mTOR is an evolutionarily conserved serine/threonine protein kinase that integrates various extracellular and intracellular signals to regulate cellular homeostasis and metabolism. In mammals, mTOR constitutes the catalytic subunit of two distinct complexes, known as mTOR complex-1 (mTORC1) and mTOR complex-2 (mTORC2). mTORC1 is a complex formed by several proteins, including DEPTOR (DEP-domain-containing mTOR-interacting protein), Raptor (regulatory associated protein of mTOR), GβL (G protein β subunit-like protein)/mLST8 (mammalian lethal with SEC13 protein 8), and PRAS40 (the 40 kDa proline-rich AKT substrate). mTORC2 is composed of mTOR, Rictor (rapamycin-insensitive companion of mTOR), Protor/PRR5 (proline-rich protein 5), GβL/mLST8, DEPTOR, and mSIN1 (mammalian stress-activated protein kinase-interacting protein 1).

mTORC1 is well known for its role in metabolism and cell growth and is influenced by various nutrients and hormonal signals, particularly the presence of amino acids, glucose, oxygen, and cholesterol availability, while mTORC2 regulates cell proliferation and survival. mTOR plays a significant role in regulating lipid metabolism, protein biogenesis, and glucose metabolism. mTOR is capable to phosphorylate serine/threonine or tyrosine residues, exhibiting dual kinase activity, and is considered part of the phosphoinositide 3-kinase (PI3K) family due to its catalytic domain, which has a similarity with lipid kinases like PI3K [35].

Frequent alterations in mTOR have been noted to play a significant role in tumorigenesis, distant metastasis, and drug resistance in human cancers such as those of the lung, breast, liver, kidney, pancreas, and prostate. The activation of the mTOR pathway has been shown to enhance tumor growth by regulating glycolysis, angiogenesis, growth factor receptor pathways, lipid metabolism, and autophagy. Therefore, mTOR is considered an important and promising target for therapeutic intervention against human malignancies.

Numerous studies have identified mTOR signaling as a key protein in regulating the aging processes across a wide range of organisms, including worms, yeast, flies, and mammals [36,37,38,39]. One of the pioneering studies to explore mTOR’s role as a regulator of the aging process reported that the absence of the mTOR homolog, known as let-363/CeTor, in the nematode Caenorhabditis Elegans causes a significantly extended mean lifespan, more than doubling its natural lifespan [40]. Later, multiple studies demonstrated that the inhibition of mTOR can also prolong lifespan in humans and numerous disease models [41]. These findings naturally sparked considerable interest in the potential for mTORi to extend lifespan. Initially, RAPA was identified as an antifungal compound derived from Streptomyces hygroscopicus, found in a soil sample collected from Easter Island, also known as Rapa Nui. Afterwards, RAPA was discovered to have antiproliferative and immunosuppressive effects because of its ability to form a gain-of-function complex with the 12 kDa FK506-binding protein (FKBP12), which binds and functions as an allosteric inhibitor of mTORC1 but not of mTORC2 [42], even though chronic administration of RAPA has been shown to inhibit mTORC2 and mediate many of the negative metabolic side effects of the drug [43]. In 2009, a study by the National Institute on Aging Interventions Testing Program (ITP) showed, for the first time in mammals, that rapamycin increased the lifespan of genetically heterogeneous mice [44].

Several mechanisms could underlie the anti-aging effects of mTORi. Firstly, mTOR is considered a key regulator of cellular proteostasis and mRNA translation is primarily regulated at the initiation step by mTORC1 via the ribosomal protein S6 kinase (S6K) and eukaryotic translation initiation factor 4E-binding proteins (4E-BPs) [45]. Some evidence has shown that the deletion or knockdown of S6K1 or translation initiation factors leads to increased lifespan in model organisms [46,47]. Conversely, other studies have demonstrated that neither mTOR inhibition by rapamycin nor deletion of S6K1 significantly slows protein translation in mice or cells [48,49]. These findings suggest that the inhibition of mTOR does not extend longevity solely by downregulating protein translation. Thus, Shin et al. revealed that targeting mTOR inhibits eIF4E-mediated cap-dependent translation with consequent activation of eukaryotic translation initiation factor 3D (eIF3D), sustaining alternative non-canonical translation mechanisms. Additionally, eIF3D-mediated protein synthesis facilitates cell phenotype switching from proliferative to more migratory cell states, suggesting that rapamycin might exert a more complex effect on protein translation [50].

SGLT-2 is an integral membrane protein, encoded by the SLC7A2 gene, which is involved in the reabsorption of almost 90% of the glucose filtered by the glomerulus and reuptake in the early proximal tubule. The sodium–glucose co-transporters family consists of 12 member proteins and related human genes, sharing a sequential molecular transport mechanism. This process involves sodium binding at the extracellular surface, opening the gate to capture outside sugar, and subsequently releasing sodium and glucose into the cell cytoplasm. Within the family, SGLT-1 and SGLT-2 have garnered the most attention due to spontaneous mutations of the respective genes and their medical use as antidiabetic treatments [51].

**Figure 1:**
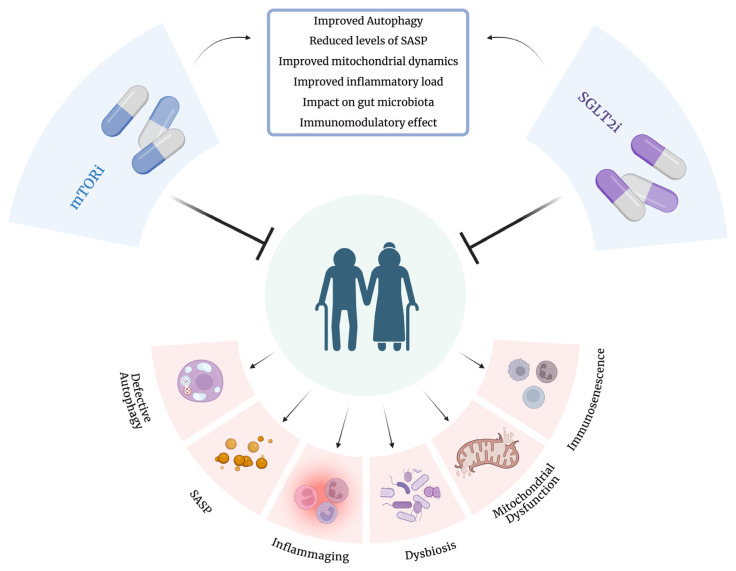
mTOR and SGLT-2 inhibitors synergistic effects. mTOR and SGLT-2 inhibitors play a significant role in many cellular processes considered hallmarks of aging. mTORi enhance autophagy, promoting the removal of dysfunctional or unnecessary cellular components, reduce the age-related increase of proinflammatory cytokines by dampening immune responses, and have a positive effect on mitochondrial dynamics. SGLT-2i improve metabolic health, which indirectly supports autophagy and reduces inflammation. Additionally, they have been shown to have potential immunomodulatory properties. Furthermore, these drugs can influence gut microbiota composition, mitigating dysbiosis, a condition linked to age-related diseases. Collectively, these effects contribute to healthier aging by targeting key cellular processes. Created with https://www.BioRender.com (accessed on 23 July 2024).

Some studies have indicated that SGLT-2i can prevent the accumulation of senescent cells [52]. These benefits seem to result from the ability of SGLT-2i to simultaneously increase nutrient deprivation signaling and decrease nutrient surplus signaling. These changes in nutrient sensing lead to reduced activation of mTOR receptors and increased expression and activity of AMP-activated protein kinase (AMPK), sirtuin 1 (SIRT1), sirtuin 3 (SIRT3), sirtuin 6 (SIRT6), and peroxisome proliferator-activated receptor γ coactivator 1-α (PGC1-α) [53]. By inducing glycosuria, SGLT-2i simulate a state of caloric loss, leading to the hepatic production of ketone bodies, primarily β-hydroxybutyrate. Moreover, their use has been shown to reduce uric acid levels, a marker of oxidative stress, and promotes gluconeogenesis and erythrocytosis [54]. Large randomized controlled trials have consistently found that increases in hemoglobin and reductions in uric acid are significant predictors of SGLT-2i’s effectiveness in reducing the risks of hospitalizations and major adverse renal and cardiac events [55]. Furthermore, a growing body of evidence suggests that amino acid deprivation seems to trigger lysosomal/autophagy degradation more than RAPA [56]. This suggests that SGLT-2 inhibitors could be considered as an additional therapy alongside RAPA for treating conditions characterized by prolonged mTOR activation and age-related diseases [57] (Figure 1 and Table 1).

### 3.1. Autophagy

Autophagy is a cellular process involved in the removal of dysfunctional or unnecessary components, such as damaged organelles and misfolded proteins, enabling cells to adapt to stress conditions. Research indicates that the body’s capacity to recycle nutrients via autophagy plays a crucial role in modulating animal lifespan [58]. mTOR is considered one of the most crucial pathways associated with the suppression of autophagy via nuclear respiratory factor-1 (NRF1) [59,60]. When the nutrients are abundant, mTORC1 could serves as a suppressor of autophagy by phosphorylating components of the autophagy process, such as the autophagy-activating kinase 1 (ULK1), autophagy-related gene 13 (ATG13), and focal adhesion kinase family-interacting protein of 200 kDa (FIP200). Contrarily, during periods of nutrient scarcity, autophagy is stimulated due to the activation of AMPK and decreased mTORC1 activity [61,62,63]. This evidence suggests an inverse correlation between autophagy and mTORC1 activation. Thus, inhibition of mTOR through RAPA has been shown to significantly elevate levels of autophagy-related proteins in animal models, together with an increase in expression of anti-aging klotho protein [64].

In lupus nephritis models, autophagy is typically inhibited by the activation of mTOR, which contributes to abnormal immune cell lineage specification in systemic lupus erythematosus. Notably, alterations in several genes that regulate autophagy, such as ATG5 and ATG7, are associated with molecular pathogenesis of the disease. RAPA induces autophagy, restores podocyte foot processes, and reduces podocyte apoptosis, resulting in decreased albumin flux and proteinuria [65].

In tumor settings, both in vivo and in vitro studies have demonstrated that RAPA significantly hyperactivates the autophagic pathway in Kaposi’s sarcoma lesions, reducing cancer cell viability, as shown by Lupinacci et al. [66]. Furthermore, previous studies have demonstrated that RAPA increases the sensitivity of PTEN mutant testicular cancer cells to radiotherapy by inducing autophagy. Additionally, a phase IB clinical study showed that RAD inhibited the growth of endometrial cancer cells and showed synergistic effects when combined with other anticancer drugs [67]. Moreover, defective autophagy has been found to promote invasion and stemness of glioblastoma cells, leading to a worse prognosis. In this context, autophagy suppression appears to be correlated with the upregulation and overactivity of mTORC1. Ferese et al. demonstrated that inhibiting mTORC1 with RAPA increases the rates of autophagy, mitophagy, and mitochondriogenesis, with these effects lasting for weeks after RAPA withdrawal [68].

Excessive mTOR activity is associated with the onset of epilepsy, with mTORC1 playing a pivotal role in the process of epileptogenesis. It has been proposed that autophagy may prevents the onset of epilepsy by modulating the balance between inhibitory and excitatory neurotransmitters, such as GABA and glutamate. The crucial role of RAPA in reducing the intensity of induced seizures in animal models has been highlighted. Clinical evidence suggests that RAPA is efficacious in patients diagnosed with tuberous sclerosis complex and has a clear anti-epileptic effect. Therefore, inducing autophagy could present an innovative therapeutic approach to epilepsy management [69].

Xing et al. explored the interplay between autophagy and osteogenic differentiation in bone marrow mesenchymal stem cells (BMSCs) using RAPA. They observed that the basal autophagy level in BMSCs decreased gradually during osteogenic differentiation. Conversely, it increased with higher concentrations of RAPA, indicating that an optimal RAPA concentration promoted BMSC osteogenic differentiation by activating autophagy [70].

Recently, mounting evidence has indicated that SGLT-2i improve autophagy in various organs, including the kidney and heart [71,72,73,74]. According to Osataphan et al., inhibiting SGLT-2 causes fasting-like and hypoxia-like transcriptional changes responsible for the activation of autophagy in overnutrition diseases [75]. In addition to activating AMPK and directly inhibiting mTORC1 through glucose excretion, SGLT-2 inhibitors also elevate the glucagon/insulin ratio, promoting hepatic gluconeogenesis. This process consumes circulating amino acids, thereby inhibiting mTORC1 and initiating autophagy [76]. In diseases associated with overnutrition, SIRT1 is suppressed in proximal renal tubules due to the increased expression of SGLT-2 induced by environmental glucose. This upregulation promotes energy uptake, lipid synthesis, and storage. Sirtuins are a family of redox-sensitive nicotinamide adenine dinucleotide (NAD)-dependent deacetylases responsible for post-translational changes of numerous proteins crucial for metabolism and cellular homeostasis and act in response to cellular glucose restriction [77]. Therefore, pharmacologically inhibiting SGLT-2 leads to increased SIRT1 expression in proximal tubules and enhances autophagy flux in these conditions [78].

Autophagy is induced under hypoxic conditions, with hypoxia-inducible factors (HIF) playing a crucial role in its enhancement. In overnutrition diseases, there is an imbalance between HIF-1α and HIF-2α: HIF-1α is overactivated by factors such as high glucose, advanced glycation end products (AGEs), mTOR, and hypoxia, whereas HIF-2α is suppressed due to SIRT1 inhibition. SGLT2 inhibitors appear to mitigate this imbalance, helping to restore the equilibrium between HIF-1α and HIF-2α, thereby promoting autophagic flux [79].

### 3.2. Inflammaging

An age-related increase of proinflammatory cytokines results in an increased expression of endothelial damage mediators and matrix remodeling, a phenomenon named “inflammaging” [80]; indeed, they release proinflammatory substances such as IL-1, IL-6, and TNF-α that are regulated by transcription factors through the mTOR network [81]. Jiang et al. showed that reducing mTORC1 activity in murine macrophages by knocking down Raptor (regulatory associated protein of TOR) lowers the expression of inflammatory genes. Conversely, activating mTORC1 signaling by downregulating its inhibitor, TSC1 (tuberous sclerosis complex1), increases glycolysis and induces a shift towards proinflammatory M1-like macrophages [82,83]. Moreover, it has been shown that the senomorphic effects of mTORi are not only related to their inhibition of the mTOR signaling pathway but also to other potential or secondary mechanisms, such as activation of the Nrf2 pathway and decreased NF-kB activity to reduce IL-1α production [84,85]. The inflammaging process results in an increased expression of proteins associated with endothelial damage, vascular smooth muscle cell proliferation, and matrix remodeling, such as intercellular adhesion molecule-1 (ICAM-1), metalloproteinases, and vascular cell adhesion molecule-1 (VCAM-1) [86]. However, recent studies suggest that RAPA treatment reduces the expression of markers of cellular senescence but has no significant effect on chronic inflammation [87].

Hyperglycemia triggers podocytes, mesangial cells, and tubule cells to release IL-6, contributing to systemic and localized subclinical inflammation, and the use of SGLT-2i has been demonstrated to reduce IL-6 levels [88]. Moreover, the binding of TNF-α to its receptor TNFR1 in podocytes increases inflammation and cytokine production in the kidney. Heerspink et al. were the first to demonstrate that canagliflozin lowers TNFR-1 levels in individuals with T2D [89]. These results were confirmed by findings from the CANVAS trial that have shown that the SGLT-2i, canagliflozin, reduces the rise in TNFR-1 and TNFR-2 levels [90], improving the inflammatory load.

### 3.3. Senescence-Associated Secretory Phenotype

Senescent cells remain metabolically active and exhibit a distinct phenotype known as the senescence-associated secretory phenotype (SASP), even though they are in a growth-arrested state. This phenotype is characterized by structural changes in the cell, such as nuclear remodeling, increased lysosomal activity, resistance to apoptosis, and alterations in the secretory profile, including the production of cytokines, proteases, and growth factors [91]. SASP can alter tissue structure and function, promoting the development of malignant phenotypes in neighborhood cells. Several studies have shown that mTOR is involved in the development of SASP. Firstly, mTOR has been demonstrated to directly influence NF-κB activity. The activation of PI3K–AKT via p38–MAPK signaling promotes mTOR activation in pre-senescent prostate cancer cells, as well as in senescent cancer cells induced by chemotherapy or radiation treatment, thereby suppressing its negative activity against NF-κB. Moreover, mTOR can modulate the expression of SASP factors post-transcriptionally by enhancing mRNA stability [92]. Additionally, previous studies have demonstrated that the mTOR signaling pathway regulates the translation of IL-1α to induce SASP [93]. Thus, inhibition of mTORC1 with mTORi can selectively block the translation of membrane-bound IL-1α, thereby inhibiting the expression and secretion of inflammatory cytokines in senescent cells. IL-1α is a key component of the autocrine amplification loops that can reduce the expression and secretion of IL-6 and IL-8 and regulate other SASP factors. The effects of RAPA on the SASP may be linked, in part, to the role of 4EBP1, a substrate of mTORC1, in regulating the phosphorylation of the RNA-binding protein ZFP36L1 during aging, inhibiting the degradation of SASP components [94,95]. Furthermore, Wang et al. showed that in Nrf2KO mouse fibroblasts, RAPA inhibited the secretory phenotype of senescent cells through an Nrf2-independent mechanism. This suggests that cell senescence is a complex process, and while RAPA uses Nrf2 to regulate cell cycle arrest, Nrf2 does not impact the production of SASP [95]. Other studies have demonstrated that low-oxygen conditions activate AMPK in senescence cells, which in turn suppresses the mTOR-NF-kB signaling loop. Therefore, treatment with hypoxia-mimetic compounds decreases SASP in cells and tissues, in aged mice [96].

Studies have demonstrated that SGLT-2i decreases the levels of circulating inflammatory molecules, including TNF-α, monocyte chemoattractant protein 1 (MCP-1), platelet endothelial cell adhesion molecule-1 (PECAM-1), VCAM-1, ICAM-1, IL-1β, and IL-6, exhibiting anti-inflammatory and antifibrotic properties [97,98]. Empagliflozin has been shown to enhance the quality of life and cardiac functional capacity in patients with heart failure and T2D. This improvement is linked to a significant reduction in IL-6 levels, suggesting that the anti-inflammatory effects of SGLT-2 inhibitors may contribute to their benefits in heart failure [99]. In a comparative study involving 32 diabetic patients, Alshnbari et al. evaluated the effects of canagliflozin and empagliflozin on inflammatory cytokines. The results indicated that a 6-month treatment with empagliflozin was effective in reducing the inflammatory cytokines IL-6 and TNF-alpha [100]. Furthermore, in a T2D model of db/db mice, treatment with dapagliflozin led to reduced expression levels of SASP in the kidney, suggesting a role for this pharmacological compound in an aging SAS-phenotype [101].

### 3.4. Immunosenescence

Aging of the immune system, also known as “immunosenescence”, involves significant alterations in both innate and adaptive immune responses, resulting in diminished immune function in the elderly. This includes dysregulation in T-cell response, impaired B-lymphopoiesis, and reduced activity of antigen-presenting cells [102]. In this setting, B and T cells showed an increased expression of proapoptotic molecules and a reduced function, which cause an increased susceptibility to cell death [103]. Research indicates that mTORi enhances the performance of the aging immune system in both mice and humans. In a recent study, CD4+ T cells from older adults were exposed to a physiological dose of RAD, demonstrating a significantly reduction of inflammatory cytokines produced by various T cell subsets, specifically by Th17 subsets [60]. Chen et al. showed that RAPA increased the production of naïve B-cell in elderly mice, leading to better immunoprotection after influenza vaccination [104]. The evidence obtained from the animal models align with the results reported by Mannick et al., who demonstrated that RAD significantly enhanced antibody titers in response to the influenza vaccine in a randomized controlled trial involving older adults [105]. Additionally, mTORi have been shown to induce a better immune response to the COVID-19 vaccine in immunosuppressed frail patients, as suggested by the increased production of vaccine-induced antibodies and by the improved cell-mediated immune response [106,107]. Thus, RAPA and RAD have been shown to act as immunomodulators; at anti-aging doses, they eliminate hyperimmunity rather than suppressing immunity and improving immune responses, not only in elderly but also in frail patients, as reported by Blagosklonny [107]. Furthermore, a recent meta-analysis showed that the immunomodulation resulting from mTORi treatment enhances the survival rate during acute infection in animal models [108,109].

Recently, studies have revealed new aspects of SGLT-2i, highlighting their potential immunomodulatory properties. Jenkins et al. demonstrated that in vitro exposure to canagliflozin inhibits T cell receptor signaling, leading to reduced mTORC1 activity in T cells, resulting in compromised metabolic reprogramming and T cell effector function. Furthermore, when CD4 T cells from patients with autoimmune disease were treated with canagliflozin in vitro, they produced fewer proinflammatory cytokines and showed reduced activation. These findings suggest that canagliflozin may reduce pathogenic T cell function in autoimmunity. Notably, SGLT-2 is minimally expressed on T cells, indicating that canagliflozin may act through an independent mechanism, which could differ among various SGLT-2 inhibitors, as evidenced by the use of dapagliflozin, which had no effect on T cells [109]. Additionally, T cells isolated from patients with immune thrombocytopenia revealed that empagliflozin increased the regulatory T cell subset while decreasing the Th1 and Th17 T cell subsets. Qin et al. noted that the administration of an mTOR agonist neutralized the impact of the SGLT-2i, indicating, once again, that SGLT-2i may influence the metabolic reprogramming of CD4 T cells through the mTOR signaling pathway [110,111]. Furthermore, canagliflozin was found to improve pathological damage in the glomeruli and enhance the expression levels of podocyte marker proteins. This was achieved by reversing the imbalance between Helper T cell 1 and 2 (Th1 and Th2) in the peripheral blood of rats with membranous nephropathy. Consequently, canagliflozin significantly inhibited the synthesis of immunoglobulin G1 in B cells and reduced the deposition of immune complexes beneath the glomerular epithelium, indicating the protective and immunomodulatory effects of SGLT-2i on kidneys [111]. Additionally, macrophages exposed to dapagliflozin exhibited a shift from inflammatory M1 macrophages to anti-inflammatory M2 phenotypes [112].

### 3.5. Dysbiosis

The microbiome is composed of several microorganisms colonizing the mucosal surfaces, gut, and skin and plays a crucial role in essential functions of the human body, including immunity, circadian rhythms, metabolism, and nutritional responses [113]. The intestinal microbiota communicates with the peripheral and central nervous systems, as well as other distant organs, impacting the overall maintenance of host health. Thus, disruption of this communication contributes to various pathological conditions, including obesity, T2D, ulcerative colitis, neurological disorders, cancer, and cardiovascular diseases [114,115].

The diversity of the microbial community within the intestinal tract is influenced by several factors, including ethnicity, lifestyle habits, dietary factors, and the environment. Moreover, as individuals age, the structure and function of this bacterial community undergoes gradual changes, ultimately resulting in a general decline in ecological diversity [116].

Gut microbes and their metabolites can modulate host metabolic and immune responses via the mTOR pathway, leading to disruptions in host physiological functions. In colon cancer, the dysregulation of gut microbiota is accompanied by the release of endotoxin lipopolysaccharide (LPS) and cathepsin K. Cathepsin K binds toll-like receptor 4 (TLR4) on macrophages surfaces to activate the mTOR pathway, enhancing the M2 polarization of tumor-associated macrophages and promoting cancer progression via the mTOR-dependent pathway [117,118]. Moreover, isolation of the Escherichia coli strain NF73-1 from the intestines of a non-alcoholic steatohepatitis patient can induce M1 macrophages phenotype in liver mice, promoting the development of NAFLD, as shown by Zhang et al. This induction involves the activation of mTOR-S6K1-SREBP-1/PPAR-α signaling, leading to a metabolic shift from triglyceride oxidation to triglyceride synthesis in NAFLD mice [119]. Liu et al. demonstrated that mTOR is involved in microbiota regulation by investigating the effects of the probiotic Clostridium butyricum (CB) on the intestinal barrier function. They showed that CB induces the upregulation of AKT/mTOR and alters the production of inflammatory cytokines, leading to a decreased production of TNF-α, IL-1β, and IL-13, and increased release of IL-10, thereby exerting a protective effect on intestinal barrier function [120]. Conversely, Lactobacillus X12 was demonstrated to suppress the proliferation of colorectal cancer cells by inhibiting mTOR and modulating cell-cycle-associated proteins [121]. Although the mTOR pathway has been implicated in the development of diseases where alterations in the microbiome have been shown to play a crucial role, such as the progression of kidney injury in diabetic nephropathy [122], myocardial injury [123], dermatitis [124], and metabolic disease [125], the direct association between alterations in gut microbiota and mTOR remains unclear. However, studies have shown that this association offers promising avenues for treatment. For example, resveratrol, another mTORi, mitigated alterations in intestinal microflora in diet-induced obese mice [126]. Additionally, the long-term use of RAPA produces modest global intestinal metagenomic changes and significant changes in some taxonomic units, such as Firmicutes, Acidobacteria, and Bacteroidetes [127]. Moreover, caloric restriction diets, associated with the suppression of the mTOR pathway, induce structural changes in the gut microbiome, increasing the abundance of Lactobacillus and other species that contribute to healthy aging [128,129].

SGLT-2i have been shown to have an impact on gastrointestinal metabolites and gut microbiota and improve overall host healthspan. Animal studies have demonstrated that SGLT-2i treatment induces changes in the gut microbiota composition and in their metabolites. Specifically, SGLT-2i have been shown to promote the growth of bacteria that produce short-chain fatty acids, which could contribute to their beneficial effects on improving insulin sensitivity, regulating energy metabolism, and reducing systemic inflammation [129]. Yang et al. investigated the impact of dapagliflozin on fecal microbiota in a T2D rat model. They showed that dapagliflozin, together with metformin, synergistically improved beneficial gut bacteria, thereby improving the composition of fecal microbiota in the management of T2D [130]. Additionally, treatment with SGLT-2i significantly increased the overall prevalence of 12 types of bacteria involved in regulating the gut microbiota balance. There were notable increases in the prevalence of short-chain fatty acid-producing bacteria with a prevalence of ruminococci, suggesting that, given that short-chain fatty acids are known to mitigate obesity, SGLT-2i may promote weight loss through their effects on gut microbiota [131]. However, Lee et al. demonstrated that diabetic animals displayed significant dysbiosis, but they observed that dapagliflozin administration induced only minor alterations in the quantity and diversity of microbial communities in diabetic animals compared to control [132].

Nevertheless, since the mechanisms and effects of these potential treatments remain unknown, it is crucial to explore other therapies related to gut microbiota in future studies.

### 3.6. Mitochondrial Dysfunction

With physiological aging, mitochondrial function declines due to several mechanisms, including the accumulation of mutations in mitochondrial DNA, impaired proteostasis which leads to the destabilization of respiratory chain complexes, decreased turnover of the organelle, and alterations in mitochondrial dynamics. This scenario compromises the role of mitochondria in cellular bioenergetics, increases the production of reactive oxygen species (ROS), diminishes ATP production, and can permeabilize the mitochondrial membranes, resulting in inflammation and cell death [133]. The mTOR pathway plays a role in the selective degradation of dysfunctional mitochondria through autophagy, a process known as mitophagy. Mitophagy is believed to maintain mitochondrial DNA (mtDNA) quality due to the removal of defective mitochondria. RAPA treatment has been shown to significantly induce mitophagy and reduce mitochondrial mutation frequency in vitro [134,135,136,137]. Bielas et al. demonstrated that mTORi significantly reduce the electron transport chain-deficient fiber abundances and lower mtDNA deletion frequency in mice. This suggest that the lifespan-extending effects of long-term RAPA treatment are due to the improved mtDNA quality [137]. Cheema et al. investigated mitochondrial myopathies, particularly MELAS (Mitochondrial Encephalopathy, Lactic Acidosis, and Stroke-like episodes), one of the most common maternally inherited mitochondrial disorders characterized by point mutations in mtDNA. They found that mitochondria from MELAS patients were fragmented, exhibited a reduction in mitochondrial membrane potential, and a decline in basal respiration. Treatment with RAPA enhanced mitochondrial respiration, increased lysosome content, and improved mitochondrial localization to lysosomes in MELAS fibroblasts, suggesting that mTORi may potentially enhance cellular health in the presence of mtDNA defects, primarily through increased lysosomal content [138].

Impaired mitochondrial turnover has been found to play a crucial role in the progression of tumors, including glioblastoma. Lenzi et al. demonstrated that the use of RAPA induces the expression of genes related to mitochondrial fission and mitophagy, such as PINK1, PARKIN, ULK1, AMBRA1, FIS1, and DRP1, along with overexpression of autophagy-related genes, leading to increased and durable mitochondrial plasticity [139].

Mitochondrial impairment plays a pivotal role in the development of Alzheimer’s disease, as evidenced by increased permeability and decreased mitochondrial membrane potential, which lead to cellular oxidative stress. Moreover, damaged mitochondria release cytochrome C, initiating the caspase cascade and inducing neuronal apoptosis. Studies show that RAPA enhances mitophagy mediated by PARKIN, an ubiquitin–ligase protein that promotes autophagic processes, facilitating the fusion of mitophagosomes with lysosomes in the hippocampus of mice. Additionally, mTORi improves learning and memory, synaptic plasticity, while reducing oxidative stress and restoring mitochondrial function [140].

Positive effects on mitochondrial dynamics were also observed during treatment with SGLT-2i. Evidence indicates that these drugs can restore mitochondrial morphology and function. Specifically, Takagi et al. found that SGLT-2i treatment is linked to the restoration of physiological levels of mitofusin 2 and optic atrophy 1, which are important factors involved in mitochondrial fusion [141]. Moreover, Zhou et al. demonstrated that empagliflozin may exert beneficial effects by inhibiting diabetes-induced mitochondrial fission through AMPK activation. By activating AMPK, empagliflozin effectively inhibited Drp1 activation, thereby reducing mitochondrial fission. This delay in microvascular endothelial cell senescence and enhancement of angiogenesis helps mitigate tissue injury [142]. Maintaining mitochondrial homeostasis requires a perfect balance between mitochondrial biogenesis and mitophagy. It has been observed that empagliflozin may utilize the mitophagy mechanism to halt mitochondrial fission in diabetes [143].

**Table 1:** Collected studies of the paragraph and their major findings.

Senescence Process	mTORi	SGLT-2i
Autophagy	Increase expression of autophagy-related proteins and Klotho protein in mice [58]	Cause fasting-like and hypoxia-like transcriptional changes responsible for the activation of autophagy in overnutrition diseases [69]
	Reduce Kaposi’s sarcoma cell viability by increasing autophagy through p75^NTR^ via EGR1 [60]	Elevate glucagon/insulin ratio, promoting hepatic gluconeogenesis, inhibiting mTORC1, and initiating autophagy [70]
	Increase sensitivity of PTEN mutant testicular cancer cells to radiotherapy and inhibit the growth of endometrial cancer cells by inducing autophagy [61]	Increase SIRT1 expression in proximal tubules, enhancing autophagy [72]
	Increase rates of autophagy, mitophagy, and mitochondriogenesis in glioblastoma cells [62]	Promote autophagy through restoration of the equilibrium between HIF-1α and HIF-2α [73]
	Reduce intensity of seizures in animal models by inducing autophagy [63]	
	Promote bone marrow mesenchymal stem cells osteogenic differentiation by activating autophagy [64]	
Inflammaging	Activate the Nrf2 pathway and decrease NF-kB activity, reducing IL-1α [78,79]	Reduce IL-6 levels [82]
	RAPA treatment has no effect on chronic inflammation [81]	Lower TNFR-1 and TNRF-2 levels in individuals with T2D [83]
SASP	The effects of RAPA on the SASP may be linked in part to 4EBP1 [88]	Decrease the levels of circulating inflammatory molecules exhibiting anti-inflammatory and antifibrotic properties [91,92]
	RAPA inhibits the secretory phenotype of senescent cells through an Nrf2-independent mechanism [89]	Reduce IL-6 levels in T2D patients [93]
		Reduce the inflammatory cytokines IL-6 and TNF-alpha [94]
		Reduce expression levels of SASP in mouse kidney [95]
Immunosenescence	Reduce inflammatory cytokines produced by various T cell subsets, specifically by Th17 subsets [60]	Inhibit T cell receptor signaling, leading to compromised T cell effector function [103]
	RAPA increase the production of naïve B-cells in elderly mice [98]	Increase the regulatory T cell subset while decreasing the Th1 and Th17 T cell subsets [104]
	RAD significantly enhances antibody titers in response to the influenza vaccine [99]	Revert the imbalance between Helper T cell 1 and 2 (Th1 and Th2) in the peripheral blood of rats with membranous nephropathy [105]
	Induce a better immune response to the COVID-19 vaccine in immunosuppressed frail patients [100]	Induce the shift from inflammatory M1 macrophages to anti-inflammatory M2 phenotype [106]
	RAPA and RAD have been shown to act as immunomodulators, at anti-aging doses [101]	
Dysbiosis	Mitigate alterations in intestinal microflora in obese mice [120]	Promote the growth of bacteria that produce short-chain fatty acids [123]
	Increase the abundance of Lactobacillus and other species that contribute to healthy aging [122,123]	Improve beneficial gut bacteria in T2D [124]
		May promote weight loss through their effects on gut microbiota [125]
Mitochondrial Dysfunction	RAPA treatment has been shown to significantly induce mitophagy and reduce mitochondrial mutation frequency in vitro [128,129,130]	SGLT-2i treatment is linked to the restoration of physiological levels of mitofusin 2 and optic atrophy 1 [135]
	Reduce the electron transport chain-deficient fiber abundances and lower mtDNA deletion frequency in mice [131]	Inhibit diabetes-induced mitochondrial fission through AMPK activation [136]
	RAPA enhanced mitochondrial respiration, increased lysosome content, and improved mitochondrial localization to lysosomes in MELAS fibroblasts [132]	Empagliflozin may utilize the mitophagy mechanism to halt mitochondrial fission in diabetes [137]
	RAPA induces the expression of genes related to mitochondrial fission and mitophagy [133]	
	Improve learning and memory, synaptic plasticity, while reducing oxidative stress and restoring mitochondrial function in Alzheimer’s disease [134]	

## 4. mTOR and SGLT-2 Inhibition on Senescence Pathways

**Figure 2:**
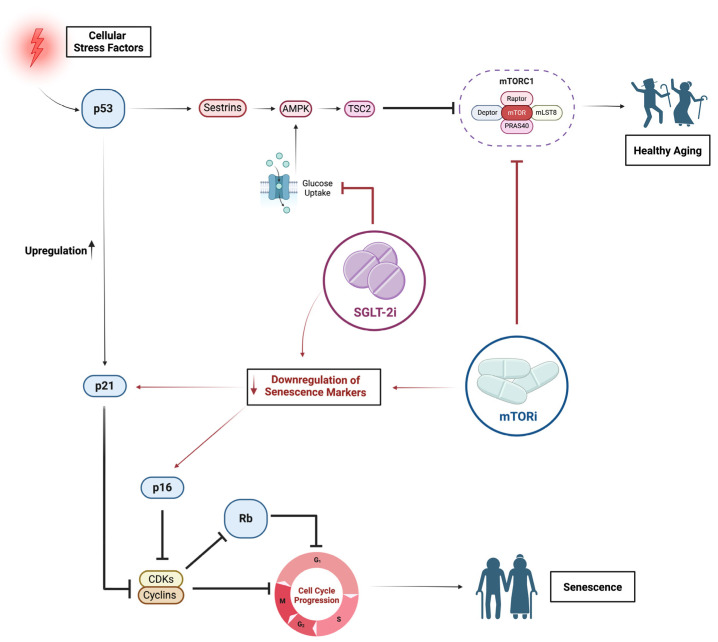
mTOR and SGLT-2 inhibition on senescence pathways. Cellular stress factors activate signaling pathways correlated with senescence (black lines). The p53–p21 and p16–Rb pathways are considered major players in the DNA damage cellular response. Modulating these pathways has been shown to increase lifespan and improve age-related processes. Both mTORi and p53 suppress mTOR through distinct mechanisms. Additionally, SGLT-2i have been shown to inhibit mTOR signaling by upregulating energy deprivation sensors like AMPK. The evidence that SGLT-2i and mTORi reduce senescence markers suggests they might have complementary mechanisms and, together, they could reduce the burden of senescence (red lines). Created with https://www.BioRender.com (accessed on 20 June 2024).

The primary cause of stress leading to cellular senescence is DNA damage, which activates the DNA damage response and the canonical p53–p21 pathway. Moreover, epigenetic alterations induce senescence primarily through the p16–Rb pathway. Studies suggest that p21 is activated early during senescence, while p16 is responsible for maintaining cellular senescence over time [144]. Both pathways are intricate, involving numerous upstream regulators, downstream effectors, and various branching routes [145,146]. These two pathways could be influenced by mTORi and SGLT-2i (Figure 2).

### 4.1. p53–p21 Pathway

P53 is recognized as a tumor suppressor gene, and it is considered a “guardian of the genome” because it maintains genome stability and cellular homeostasis. Notably, it is considered the most frequently mutated gene in cancer. Activation of p53 is promoted in response to cellular stress factors, such as DNA damage, oncogene activation, ribosomal stress, and hypoxic insults, leading to cell cycle arrest, metabolic changes, DNA repair, and induction of several cell death pathways. The cellular outcome in response to DNA damage is influenced not only by the absolute levels of p53 protein but also by the temporal fluctuations in p53 levels, which play a significant role in determining cell fate. Prolonged activation of p53 has been shown to result in irreversible cell cycle arrest and senescence [147]. p21, the first identified transcriptional target for p53, is a 21 kDa protein encoded by the CDKN1A gene and belongs to the Cip/Kip family of cyclin-dependent kinase inhibitors (CDKIs), alongside p27 and p57. It can inactivate all CDKs, thus inhibiting cell cycle progression. p21 exhibits a dual role in cell cycle regulation depending on its expression levels. At high concentrations, p21 inhibits the kinase activity of cyclin D–CDK4/6 complexes, resulting in the inhibition of cell cycle progression. In contrast, at low concentrations, p21 functions as an assembly factor for the cyclin D–CDK4/6 complex, facilitating its activation and consequently promoting cell cycle progression [148,149,150]. p53 and mTOR mutually influence each other, and coordinated regulation of both pathways plays a pivotal role in maintaining cell homeostasis. p53 controls the mTOR pathway through the transcription of multiple negative regulators of mTORC1, such as REDD1 (regulated in development and DNA damage response 1), LKB1 (liver kinase B), AMPK, tuberous sclerosis complex 2 (TSC2), and Sestrin-1 and -2. Moreover, some microRNAs (miRNA), specifically members of the miRNA-34 family, have been recognized as transcriptional targets of p53. In prostate cancer, overexpression of miR-34a upregulates the phosphorylation of mTOR. Furthermore, it has been reported that cytosolic p53 could regulate autophagy via the AMPK–mTOR axis [151]. Conversely, mTOR might regulate p53 activity both before and after translation by influencing protein synthesis and MDM2, an oncoprotein that acts as an inhibitor of p53 transactivation. The MDM2 protein can promote the translocation of p53 from the cell nucleus to the cytoplasm, leading to its rapid degradation [152].

Christy et al. showed that mTORi and p53 each suppress mTOR through distinct mechanisms, suggesting the possibility of synergistic effects. They demonstrated that p53 enhanced the efficacy of rapamycin in extending the lifespan of mice, suppressing the SASP induced by ionizing radiation and increasing the levels of amino acids and citric acid in mouse embryonic stem cells, thereby promoting mitochondrial respiration [153]. Furthermore, RAD has been demonstrated to significantly increase the oxygen consumption rate and respiratory control ratio, indicative of enhanced mitochondrial function and biogenesis. Additionally, RAD reduces circulating levels of pentraxin-3 (PTX3) and downregulates p21, two of the principal markers of inflammaging and senescence, in human peripheral blood mononuclear cells (PBMCs) [154].

Currently, human studies examining the combination of SGLT-2i and mTORi are still limited to the evaluation of the safety and efficacy of SGLT-2i in transplanted or in cancer patients. Some reports have shown that dapagliflozin and canagliflozin are able to inhibit the cancer cell proliferation blocking cell cycle in the G1/G0 phase by targeting AMPK/mTOR and inducing apoptosis [155]. Moreover, SGLT-2i have demonstrated to be associated with reduced mTOR signaling and significant transcriptional changes across all tubular segments in the kidney, leading to a mitigation of perturbed cellular metabolic profiles in T2D, as stated by Shaub et al. [156]. According to that research, the use of dapagliflozin in cultures of primary human renal tubular epithelial cells has been shown to prevent high-glucose-induced upregulation of p21, IL-1β, and IL-8 production [157].

Glucose tolerance declines with age. Overactivation of the mTOR pathway activates S6K1 and phosphorylates signaling adapter protein insulin receptor substrate 1 (IRS-1), impairing insulin-induced phosphoinositide 3-kinases (PI3K) activation and consequently increasing insulin resistance in human muscle. While RAPA treatment increased glucose turnover under induced peripheral hyperinsulinemia, it did not affect glucose turnover under low peripheral insulin conditions [158]. The catabolic-like state induced by SGLT-2i activates a fasting-like metabolic state and reduces insulin signaling due to lower glucose levels. This metabolic shift leads to increased ketogenesis, which has shown promising effects on longevity by upregulation of energy deprivation sensors, such as AMPK and SIRT1, and downregulation of nutrient sensors, such as mTOR and insulin/insulin-like growth factor-1 (IGF1) [159]. Moreover, it has been shown that the inhibition of not only p21, but also p53, can improve vascular dysfunction and suppress the development of senescent-related disease. Additionally, it has been reported that the accumulation of senescent cells in obese visceral adipose tissue leads to increased inflammation and insulin resistance. Removing p53 from adipose tissue has been shown to protect against diet-induced metabolic dysfunction. Katsuumi et al. showed that canagliflozin significantly elevated the plasma level of 5-aminoimidazole-4-carboxamide-1-β-D-ribofuranoside (AICAR), a metabolite known for activating negative regulators of mTORC1, such as AMPK, extending the lifespan of mice with premature aging and enhancing the immune-mediated clearance of senescent cells by reducing the expression of programmed cell death-ligand 1 (PD-L1) [160]. In cancer settings, SGLT-2i has been shown to inhibit glucose uptake, glycolysis, and AKT/mTOR signaling activation, while increasing AMPK expression in thyroid cancer cells, which resulted in decreased tumor cell proliferation and increased apoptosis [161]. Additionally, SGLT-2i are known to inhibit mTORC1, while their impact on mTORC2 remains unknown [162,163]. This may suggest that their utilization could be beneficial in mitigating the adverse metabolic effects caused by the inhibition of mTORC2 by chronic use of mTORi.

### 4.2. p16–Rb Pathway

The p16–Rb pathway controls G1 to S transition, one of the four critical checkpoints involved in progression of the cell cycle. p16 is a 16 kDa protein that directly binds to CDK4/6, blocking the formation of cyclin D–CDK4/6 complexes. This prevents the phosphorylation of Rb, an important tumor suppressor gene, thus inhibiting the transcription of E2F1 target genes, which are essential for the G1/S transition. Rb and p16 influence each other in a feedback loop: phosphorylation of Rb leads to increased p16 expression, which inhibits CDK4/6, resulting in higher levels of hypophosphorylated Rb, which causes decreased p16 expression. Additionally, p16 expression has been found to be elevated independently of telomere shortening and in Rb-negative cells, indicating the presence of other regulatory mechanisms [164]. In an interventional trial involving participants over 40 years old, with signs of age-related photoaging and dermal volume loss but without major morbidities, the topical application of RAPA to inhibit the mTOR pathway resulted in increased collagen VII protein levels and a significant reduction in p16 protein levels. This was accompanied by clinical and histological improvements in skin tissue [165]. Guzmán et al. demonstrated that mouse pancreatic islets exposed to glucotoxic conditions exhibit increased senescence-associated β-galactosidase activity and elevated p16 protein levels. However, low concentrations of RAPA were shown to prevent the rise of these senescence markers and reduce the insulin hypersecretion induced by glucotoxicity [166]. Moreover, RAPA is also considered an important immunosuppressive drug due to its impact on a wide range of immune cells. Thus, in 1999, it was approved as an immunosuppressant for use after organ transplantation. Based on evidence that links interstitial fibrosis and tubular atrophy, a leading cause of kidney allograft dysfunction, to premature cellular senescence, Hoff et al. demonstrated that RAPA treatment in a rat kidney transplantation model reduced p16 expression and mRNA transcripts of the proinflammatory cytokines associated with the SASP, in the tubular, interstitial, and glomerular compartments, within the first two days of starting the mTORi [167,168].

Sugizaki et al. investigated the impact of SGLT-2i treatment in a severe diabetes model by feeding diabetic mice a high-fat diet. They demonstrated that, compared to insulin-treated mice, SGLT-2i treatment reduces p16 levels in adipose cells and diminishes the number of senescent cells. Furthermore, this treatment was linked to decreased markers of oxidative stress and an increased production of antioxidants [168]. In addition, dapagliflozin was observed to prevent the advancement of diabetic kidney disease in an animal model by inhibiting cellular senescence and oxidative stress. This effect was achieved through the reduction of senescent markers, including p16 [169].

Empagliflozin treatment was found to improve the metabolic profile in mice by lowering blood glucose levels, total cholesterol, and triglycerides. Furthermore, Western blot analysis assessing the aortic levels of senescence markers revealed that empagliflozin reduced levels not only of p53 and p21 but also of p16 [170].

## 5. Conclusions

In the literature, there are few direct studies exploring the combined effects of mTOR and SGLT-2 inhibitors on aging. However, the individual benefits on senescence-related processes suggest that they might have complementary mechanisms that could be synergistic due to the combination of mTOR pathway inhibition and SGLT-2i beneficial effects in the context of age-related processes and diseases.

Their use could be accompanied by potential side effects and limitations in clinical applications. Although SGLT-2i have shown similar efficacy and safety profiles in older-versus-younger patients, with their primary focus on managing T2D and cardiovascular and renal health, the direct impact on cellular senescence remains less explored. Therefore, while evidence regarding their use in aging populations is still limited, there are concerns that they might be associated with adverse events. Specifically, the incidence of urinary tract infections, volume depletion, renal-related adverse events, and risk of hypoglycemia could be increased in the elderly. Nevertheless, the use of SGLT-2i has been associated with a favorable benefit–risk balance in elderly patients [171].

The use of mTORi is also associated with potential limitations and side effects. It is known that RAPA has been linked to an increased risk of developing new-onset T2D, likely due to a combination of insulin resistance and dysfunctional insulin secretion. Moreover, since mTOR plays a crucial role in lipid metabolism, dyslipidemia has been reported to have a high prevalence among patients on mTORi. In this context, the use of mTORi and SGLT-2i might be synergic for better glycemic control. Furthermore, mTORi increases the incidence of proteinuria due to increased glomerular permeability and subsequent podocyte injury [172]. Recent trials suggest that SGLT-2i could be useful in reducing proteinuria in kidney disease patients [173].

Further research is needed to elucidate the mechanisms through which SGLT-2i and mTORi influence cellular senescence. Genetic background, sex, pre-existing health conditions, and lifestyle may influence the efficacy and safety of these drugs; therefore, optimizing their use through long-term clinical trials, safety studies, and research focused on how these drugs interact with each other at molecular levels could allow for the development of personalized medicine strategies tailored to individual patient characteristics to identify frail patients who are most likely to benefit from the combined use of these drugs.
